# Raman spectroscopy enables non-destructive quantification of nitrate in C3 and C4 plants

**DOI:** 10.3389/fpls.2026.1858997

**Published:** 2026-07-27

**Authors:** Savitha Dhandapani, Jayapal Praveen Kumar, Alice Mei Xien Tan, Bhushan Vishal, Valiya Nadakkakath Agisha, Ekta Jain, Shaik Anwar Ahamed Nabeela Nasreen, Ganga Cheerlavancha Sravanthi, Nam Hai Chua, Gajendra Pratap Singh, Rajeev J. Ram, Bong Soo Park

**Affiliations:** 1 Temasek Life Sciences Laboratory, National University of Singapore, Singapore, Singapore; 2 Disruptive & Sustainable Technologies for Agricultural Precision, Singapore-MIT Alliance for Research and Technology, Singapore, Singapore; 3 Research Laboratory of Electronics, Massachusetts Institute of Technology, Cambridge, MA, United States

**Keywords:** C3 plants, C4 plants, nitrate quantification, nitrogen deficiency, non-destructive sensing, Raman spectroscopy, spectral biomarkers

## Abstract

Accurate assessment of plant nitrate status is critical for growth and productivity, yet early and non-destructive quantification remains challenging. Although Raman spectroscopy has been used to detect nitrate deficiency in plants, quantitative estimation of nitrate concentration from Raman spectra has not been demonstrated. Here, we evaluated whether Raman spectroscopy can be used to quantitatively predict leaf nitrate concentrations during early nitrogen stress. Two-week-old Pak Choi (C3) and Amaranthus (C4) plants were subjected to nitrate deprivation for 1–3 days, and Raman spectra were collected and correlated with nitrate concentrations determined by biochemical assays. A strong linear relationship was observed between nitrate concentration and the intensity ratio of the nitrate-associated Raman peak at 1046 cm^-^¹ to the neighboring 1067 cm^-^¹ peak. This relationship was consistent among plants of the same species and across different levels of nitrogen deficiency. Linear regression models achieved root-mean-square errors of 101 µg g^-^¹ fresh weight (FW) in Pak Choi (~7% of nitrate under sufficient nitrogen) and 32 µg g^-^¹ FW in Amaranthus (~19%), closely matching biochemical measurements and revealing species-specific nitrate dynamics. These findings demonstrate that Raman spectroscopy enables rapid, non-destructive, and quantitatively reliable estimation of leaf nitrate levels during early nitrogen stress, providing a promising platform for precision nutrient management and real-time plant phenotyping.

## 1 Introduction

Recent advances in non-invasive plant diagnostics have highlighted Raman spectroscopy as a promising tool for early detection of plant stress and physiological changes (Farber et al., 2019; Mandrile et al., 2019; Roy and Prasad, 2022). By probing molecular vibrations, Raman spectroscopy enables identification of biochemical alterations associated with diseases and nutrient deficiencies with minimal sample preparation, supporting rapid and potentially in-field applications (Zhao et al., 2020; Huang et al., 2020). It has been successfully applied for early detection of plant diseases and metabolic stress responses prior to visible symptom development (Zhao et al., 2020; Parlamas et al., 2022; Mandrile et al., 2022). The development of portable Raman systems further enhances its utility for real-time plant health monitoring and field-deployable phenotyping (Gupta et al., 2020).

Beyond disease diagnostics, Raman spectroscopy has also been increasingly explored for identifying nutrient deficiencies, including nitrogen stress. However, most studies rely on qualitative classification of stress states rather than quantitative estimation of metabolite concentrations in intact plant tissues (Altangerel et al., 2017; Sanchez et al., 2019; Huang et al., 2020; Sanchez et al., 2020; Roy and Prasad, 2022).

In contrast, regression-based chemometric approaches are widely used in analytical sciences for quantitative spectral interpretation in complex biological matrices (Esmonde-White et al., 2022; Abu-Absi et al., 2011; Yousefi-Darani et al., 2022). More recently, data-driven Raman approaches have been applied in plant systems to characterize stress-induced biochemical changes (Awasthi et al., 2025; Tripathi et al., 2025; Awasthi et al., 2026). However, robust estimation of absolute metabolite concentrations in intact plant tissues remains limited.

In this study, we develop and validate a Raman spectroscopy-based regression framework for estimating absolute nitrate concentration in intact plant tissues during the early stages of nitrogen deficiency. We examined two leafy vegetables with contrasting photosynthetic and nitrogen utilization strategies: the C3 species Pak Choi (*Brassica rapa* var. *chinensis*) and the C4 species Amaranthus (*Amaranthus tricolor* L.). C3 and C4 plants differ in their photosynthetic pathways and nitrogen use efficiency, with C4 species generally exhibiting enhanced nitrogen utilization due to reduced photorespiration (Sage and Pearcy, 1987; Taylor et al., 2011; Osborne and Sack, 2012; Taylor et al., 2014). By comparing Raman spectral responses under nitrogen-sufficient and nitrogen-deficient conditions, this study evaluates the robustness of quantitative nitrate prediction across species with distinct physiological characteristics. We hypothesize that Raman spectral signatures acquired from intact plant tissues are linearly related to the leaf-nitrate concentration and that this relationship holds across different plants of the same species grown under the similar conditions but with different degrees of nitrate deficiency. This relationship can be used to calibrate regression-based models to enable accurate, non-destructive quantification of absolute nitrate concentrations across both C3 and C4 plant systems under early nitrogen stress.

## 2 Materials and methods

### 2.1 Experimental design

The experimental workflow is summarized in **Supplementary Figure 1** and included leaf sampling, Raman spectral acquisition (830 nm excitation), biochemical analyses, and regression-based modeling.

Plants were grown under nitrogen-sufficient (+N) conditions and subjected to nitrogen-deficient (−N) treatment for 1, 2, or 3 days to induce early nitrogen stress. Six biological replicates (six independent seedlings) were used per treatment per time point. Hence, a total of 36 Pak Choi plants and 36 Amaranthus plants were measured by Raman spectroscopy and by biochemical nitrate assay.

To account for biological and spatial variability, a hierarchical sampling strategy was implemented. From each seedling, the second true leaf was selected for analysis, and three leaf discs were prepared per leaf. Raman spectra were collected from each disc at distinct positions, providing technical replicates within each biological replicate. This design enabled variance estimation at multiple levels, including plant-to-plant biological variability and within-leaf spatial heterogeneity. All plants were selected at a similar developmental stage at the beginning of treatment to minimize variation due to growth differences. The number of biological replicates was constrained by the requirement for controlled growth conditions under uniform nitrogen treatments in a growth chamber, where environmental uniformity was prioritized over large-scale plant numbers to ensure experimental consistency. Despite the limited sample size, consistent spectral and biochemical trends were observed across biological replicates and treatment conditions, supporting the robustness of the approach.

### 2.2 Plant growth

Seeds of Pak Choi (*Brassica rapa* var. *chinensis*) and Amaranthus (*Amaranthus tricolor* L.) were surface-sterilized and germinated on Murashige and Skoog medium supplemented with 1% (w/v) sucrose and MES buffer (pH 5.6). After overnight stratification at 4 °C, seeds were transferred to controlled growth conditions (16 h light/8 h dark photoperiod, 60% relative humidity, light intensity 100 µmol m^-^² s^-^¹). Growth temperatures were maintained at 22 °C for Pak Choi and 25 °C for Amaranthus.

Seven-day-old uniform seedlings, selected based on comparable developmental stage, visual morphology, and similar shoot and root growth, were transplanted onto six-hole tray lids placed over plastic containers (34 cm × 26 cm × 7.5 cm) containing 3 L of static modified Hoagland nutrient solution. The nutrient solution consisted of 2.5 mM Ca(NO_3_)_2_, 2.5 mM KNO_3_, 1 mM MgSO_4_, 0.75 µM NaFeEDTA, 0.25 mM KH_2_PO_4_, 50 µM H_3_BO_3_, 0.075 µM (NH_4_)_6_Mo_7_O_24_, 2 µM ZnSO_4_, 10 µM MnCl_2_, 1.5 µM CuSO_4_, 0.2 µM CoCl_2_, and 0.5 mM MES buffer, adjusted to pH 5.6 with an electrical conductivity of 2.4 dS m^-^¹. Nitrogen treatments were initiated two weeks after transplantation. Control plants (+N) received complete nutrient solution, whereas nitrogen-deficient plants (−N) were supplied with nitrate-free solution in which nitrate salts were replaced with equivalent chloride salts to maintain ionic balance.

Leaf samples (leaf #2) were collected at 24, 48, and 72 h after treatment initiation. Raman spectra were acquired from three positions at the leaf tip, followed by harvesting of the same tissue for biochemical analyses.

Total chlorophyll content was determined from leaf discs extracted in 80% acetone at 4 °C for 24 h in darkness. Chlorophyll concentration was calculated on a fresh weight basis according to Porra et al. (1989).

### 2.4 Nitrate quantification

Leaf nitrate content was determined according to Cataldo et al. (1975). Briefly, 100 mg of leaf tissue was homogenized in 1 mL deionized water and incubated at 100 °C for 20 min. Ten µL of the supernatant were mixed with 40 µL of 5% (w/v) salicylic-sulfuric acid and incubated at room temperature for 20 min. Subsequently, 950 µL of 8% NaOH was added, followed by incubation at room temperature for an additional 20 min. Absorbance was measured at 410 nm to quantify nitrate content.

### 2.5 Raman spectroscopy

Raman spectra were acquired from plant leaf samples using an 830 nm excitation system equipped with a sample holder incorporating a 100 µm thick fused silica window. An aspheric lens (depth of focus >1 mm) was used to focus the excitation beam and collect Raman signals across the leaf cross-section.

A fiber-coupled 830 nm laser (Innovative Photonic Solutions, USA) delivered ~100 mW of power to the sample via a 105 µm core multimode fiber. The beam was collimated and passed through a Semrock MaxLine 830 nm laser line filter (Semrock Inc., USA) to suppress amplified spontaneous emission and background signals. The filtered light was directed into the excitation path using a Semrock long-pass dichroic filter, while Rayleigh-scattered light was attenuated using an F#-matching lens prior to detection.

Raman spectra were collected using a Kymera 328i spectrograph (Andor, UK) equipped with a 600 g/mm grating.

For each biological replicate (individual plant), the second true leaf from the bottom (Leaf #2) was excised immediately prior to Raman analysis. From each excised leaf, three leaf discs were prepared and analyzed individually (one disc at a time) to minimize variability arising from leaf curvature and spatial heterogeneity.

Each disc was immediately placed on the Raman sample platform and positioned under the objective for spectral acquisition. During measurements, the samples were analyzed under controlled dark conditions; room lights were switched off during spectral acquisition to minimize potential interference from ambient light. For each leaf disc, Raman spectra were collected from a defined central region, with five spectra acquired per disc using a 10 s integration time. After each measurement, room lighting was restored and either a different disc from the same leaf or a fresh sample from the next biological replicate was positioned for subsequent acquisition.

All spectra were averaged at the disc level following preprocessing. This hierarchical sampling design (biological replicate → leaf → disc → spectra) was used to account for both biological variability and within-leaf spatial heterogeneity while maintaining consistency across all measurements.

### 2.6 Data collection and pre-processing

Cosmic ray artifacts were removed, and spectra were smoothed using a Savitzky–Golay filter (degree 2, window size 5) implemented in Python (SciPy). Fluorescence background was corrected using positive residual polynomial subtraction (Lieber and Mahadevan-Jansen, 2003), and Raman shift calibration was performed using a polystyrene standard (Creely et al., 2005).

Raman peaks provide unique vibrational fingerprints that facilitate the rapid screening of plant chemical profiles. These specific vibrational bands are given in **Supplementary Table 1**. For nitrate estimation, the intensity ratio between the nitrate peak at 1046 cm^-^¹ (Gardiner et al., 1973; Xia et al., 2025) and a neighbouring band at 1067 cm^-^¹ was calculated. To further evaluate spectral features associated with plant nitrogen status, principal component analysis (PCA) was performed using pre-processed Raman spectra obtained from nitrogen-sufficient and nitrogen-deficient plants. Loading plots of the principal components were examined to identify spectral regions contributing most strongly to treatment-related variation. The 1067 cm^-^¹ peak was used as an internal reference due to its relative stability across treatments, consistent with previous studies (Gupta et al., 2020). The intensity ratio (1046/1067 cm^-^¹) was subsequently used as the input feature for predictive modelling.

### 2.7 Model development: predicting the nitrate content

Reference nitrate concentrations obtained from biochemical assays were used as ground truth for training prediction models. A simple linear regression model, as given in Equation (1), was constructed using the Python statsmodels.api library to predict absolute nitrate concentration from the preprocessed Raman peak ratio (1046/1067 cm^-^¹):

(1) $$C_{i}=\;\theta_{0}+\theta_{1}I_{i}+\text{ℰ}$$

where $C_{i}$ is the predicted nitrate concentration for sample i, $\theta_{0}$ and $\theta_{1}$ are the regression intercept and slope, $I_{i}\;$is the preprocessed peak ratio, and ϵ is the residual error term (Zuur and Ieno, 2016).

Model performance was evaluated using biochemical assay measurements as reference values. To assess robustness with respect to plant identity and stress condition, multiple models were trained using different subsets of the dataset.

Two cross-validation strategies were applied (Yates et al., 2023). First, a holdout approach was implemented by randomly excluding all data from two plants per day under each stress condition (12 plants for Amaranthus and 12 plants for Pak Choi), while training the model on the remaining 22 plants for Amaranthus and 20 plants for Pak Choi. Second, a leave-one-day-out (LODO) strategy was used, where all measurements corresponding to a single experimental day were excluded from training and used for testing. Each daily dataset comprised 5 or 6 nitrogen-deficient plants and 5 or 6 nitrogen-sufficient plants, enabling evaluation of temporal robustness of the model.

### 2.8 Statistical analysis and data visualization

Statistical analyses for biochemical assay data (nitrate content, total chlorophyll content, and leaf area measurements) were performed using Microsoft Excel. Data are presented as mean ± standard deviation. Comparisons between +N and −N treatment groups were conducted using a two-tailed Student’s *t*-test, with statistical significance defined as *p* < 0.05*, p*  < 0.01, and *p*  < 0.001.

Linear regression analysis of the relationship between Raman peak ratio and nitrate concentration was performed using the Python statsmodels.api library. Model parameters were estimated using least-squares regression, and statistical significance of the regression was evaluated using t-tests on the regression coefficients and an F-test for overall model significance.

Median values from biochemical assays and Raman peak ratios were used to reduce the influence of outliers.

Invariance of the regression model on the particular subset of plants used for regression and on the particular stress conditions was established by performing the cross-validation procedure described above. The regression models constructed with each subset of data were used to predict the nitrate concentration for the plants that were ‘left out.’ The error between the prediction and the measured values is calculated both as a coefficient of determination (R^2^) and as a square root of the mean square error (RMSE). The R^2^ value quantifies the proportion of variance explained by the model, while the RMSE provides a measure of the average prediction error of nitrate concentration. Cross-validation is not used to establish statistical significance of any particular regression model (pair of linear regression parameters). This analysis is presented to show the independence of the linear regression model on the subset of specific plants and on the degree of nutrient limitation.

**Figure 1:**
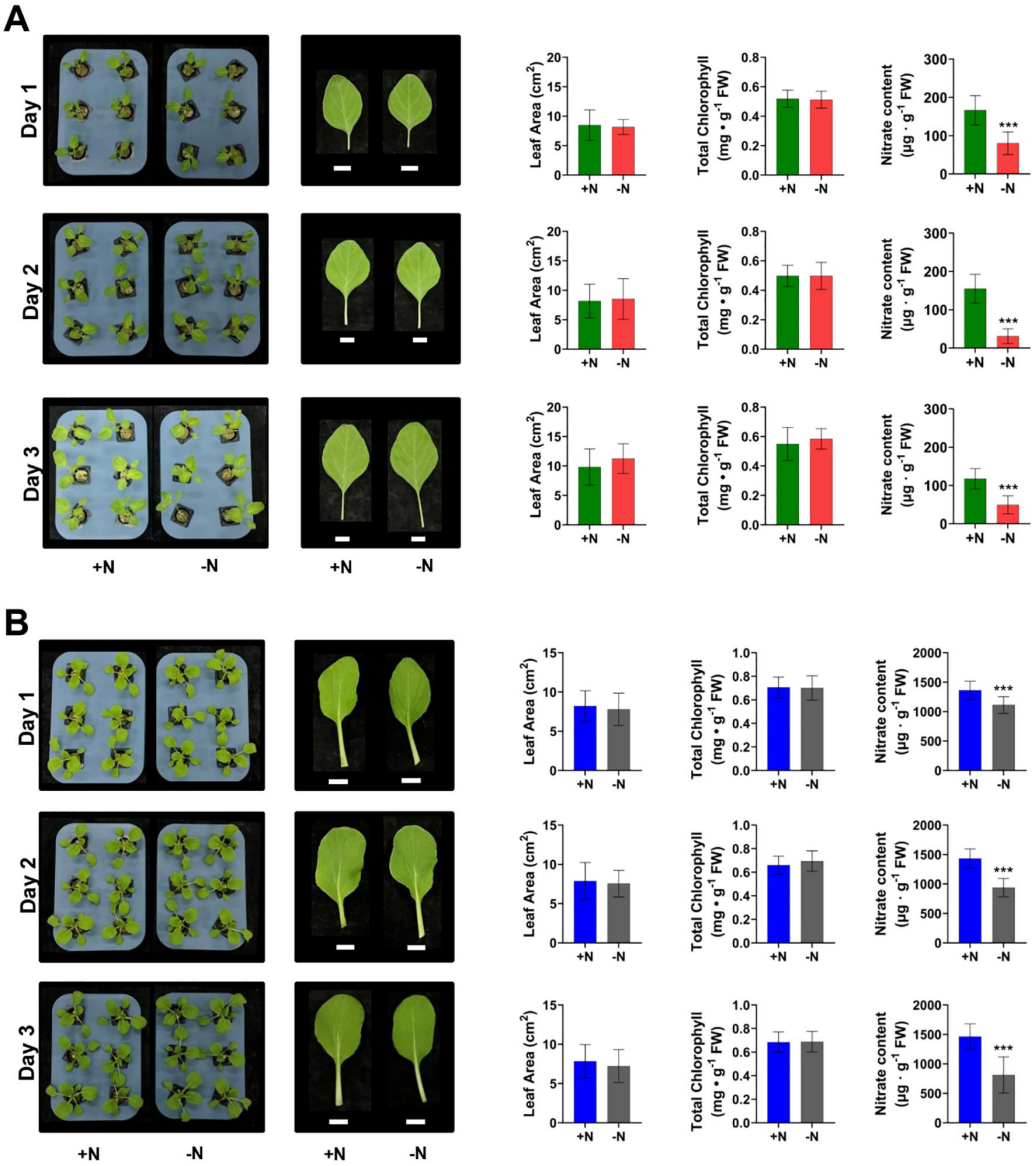
Early nitrogen-treatment responses in hydroponically grown Amaranthus and Pak Choi plants. From left to right: representative images of 2-week-old Amaranthus **(A)** and Pak Choi **(B)** plants in hydroponic trays on Day 1, Day 2, and Day 3 under +N and –N treatments; corresponding images of leaf #2 (counted from the base) for each day, scale: 1cm; the average area of leaf #2; total chlorophyll content in leaf #2; and total nitrate content in leaf #2. Statistical significance was assessed using Student’s *t*-test (***p < 0.001, **p < 0.01, *p < 0.05). Exact *p*-values are provided in **Supplementary Table 2**.

Graphical outputs in **Figure 1** were generated using GraphPad Prism version 10.3 (GraphPad Software, San Diego, CA, USA), while Raman-based regression plots and associated analyses were generated using Python.

## 3 Results and discussion

### 3.1 Nitrogen status and visual symptoms

Raman spectroscopy enabled assessment of nitrogen-dependent biochemical responses in Pak Choi (C3) and Amaranthus (C4) under +N and −N conditions. Across days 1–3, neither species exhibited visible morphological changes or chlorophyll reduction (**Figures 1A, B**), indicating that early nitrogen limitation remains visually undetectable (Huang et al., 2020).

Despite the absence of visible symptoms, nitrate concentrations declined rapidly following nitrogen withdrawal (**Figures 1A, B**). In Amaranthus, +N plants contained 117–167 µg g^-^¹ FW, while −N plants decreased from 80 µg g^-^¹ FW (Day 1) to 49 µg g^-^¹ FW (Day 3), representing an approximately 39% reduction. In Pak Choi, +N plants maintained higher nitrate levels (1362–1464 µg g^-^¹ FW), while −N plants decreased from 1113 µg g^-^¹ FW to 812 µg g^-^¹ FW over three days (≈27% reduction). These results demonstrate that nitrate depletion occurs within 24 h of nitrogen withdrawal in both species despite the absence of visible phenotypes.

The observed interspecies differences are consistent with contrasting nitrogen-use strategies. C4 plants typically exhibit higher nitrogen-use efficiency due to reduced Rubisco demand and altered carbon–nitrogen coupling, resulting in lower foliar nitrate accumulation compared with C3 species (Ghannoum et al., 2011; Leegood, 2002; Sage and Pearcy, 1987). This physiological contrast provided a robust system for evaluating Raman-based nitrate quantification across divergent metabolic backgrounds.

### 3.2 Raman detection of nitrogen deficiency and regression modelling of nitrate content

**Figure 2:**
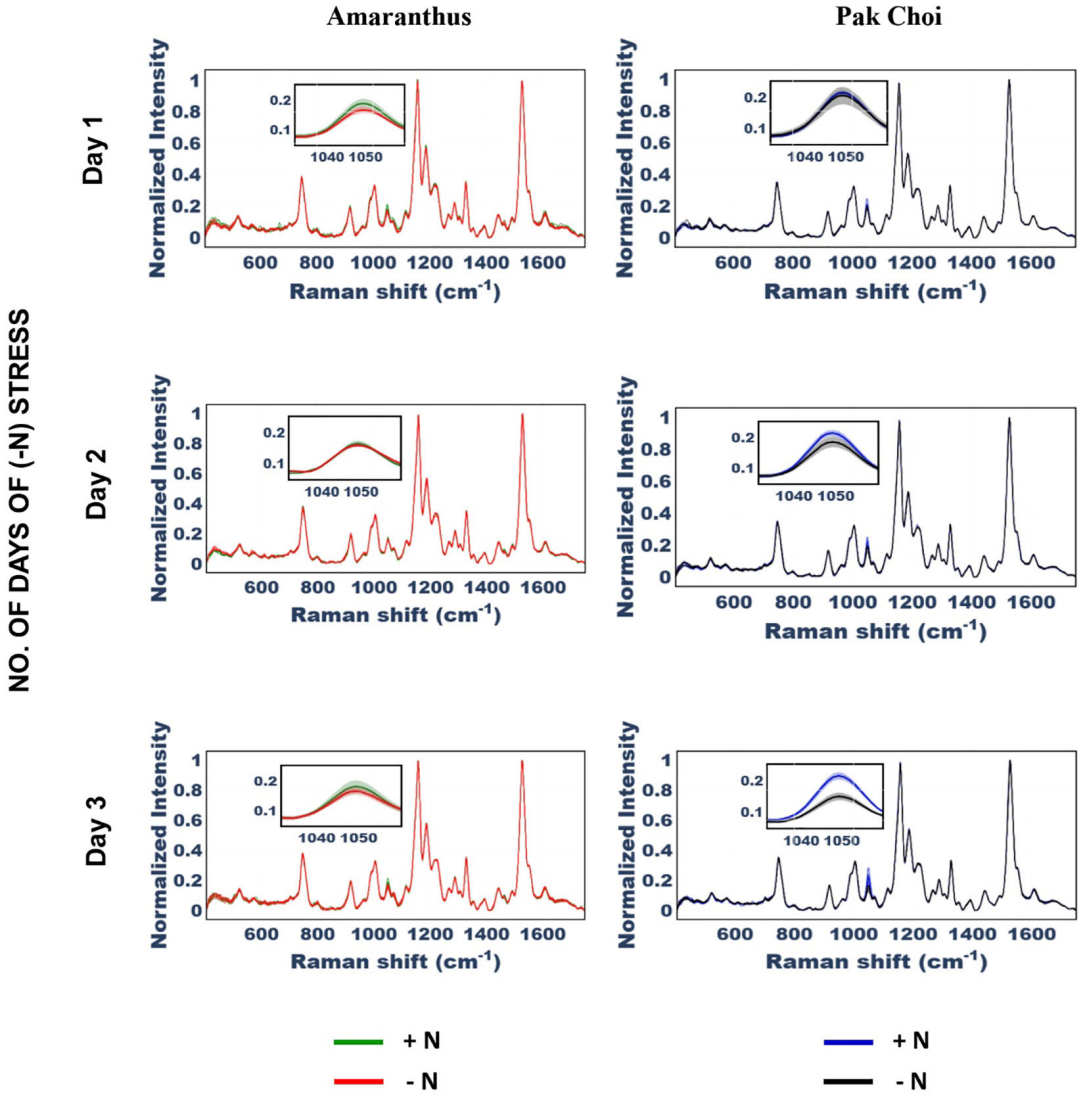
Time-series Raman spectra of Amaranthus (left panels) and Pak Choi (right panels) under nitrogen deficiency (−N) stress. The top panels represent plants subjected to 1 day of −N stress, the middle panels represent 2 days of −N stress, and the bottom panels represent 3 days of −N stress. In each panel, Raman spectra of −N-treated plants are compared with those of nitrogen-sufficient (+N) plants. Enlarged views of the nitrate peak region are included as insets to highlight changes in nitrate-associated Raman signals over the time course.

Time-series Raman analysis revealed early biochemical responses to nitrogen deprivation. After pre-processing (**Figure 2**), the nitrate-associated Raman band at 1046 cm^-^¹ decreased under −N conditions, while the 1067 cm^-^¹ band remained stable and was used as an internal reference. This normalization reduced variability arising from laser fluctuations and optical alignment. Detectable spectral differences emerged as early as 24 h post-treatment (**Figure 2**, Day 1), consistent with prior studies (Gupta et al., 2020; Huang et al., 2020).

Principal component analysis (PCA) of the Raman spectra further supported these observations. The PC1 loading revealed a prominent contribution at 1046 cm^-^¹ (**Supplementary Figure S2**), indicating that variation in this region strongly contributed to separation between +N and −N samples and supporting its association with nitrate-related biochemical changes.

Nitrogen deficiency is known to induce a wide range of biochemical and metabolic changes in plants beyond nitrate depletion. Prolonged nitrogen limitation has been associated with reductions in chlorophyll and carotenoid content, alterations in amino acid and protein metabolism, accumulation of carbon-rich secondary metabolites such as phenolics and flavonoids, and modifications to cell wall carbohydrate composition. These physiological responses can influence multiple Raman spectral regions, including chlorophyll-associated bands, C–H stretching vibrations, amide bands related to proteins, and carbohydrate-associated peaks. Previous Raman studies have demonstrated that such spectral features can serve as indicators of plant nutrient status and stress responses. In the present study, however, the analytical framework was intentionally focused on the nitrate-specific Raman band at 1046 cm^-^¹ and its ratio to the relatively stable 1067 cm^-^¹ reference band. The ability of this targeted approach to accurately predict nitrate concentrations, as validated by biochemical assays, suggests that the selected spectral feature provides a robust indicator of nitrate status despite the complex biochemical background of plant tissues.

Linear regression models were developed to predict quantitative nitrate concentration from the Raman peak ratio (1046/1067 cm^-^¹). The nitrate prediction results are given in **Table 1**, while the relationship between the Raman peak ratio and model-predicted nitrate concentrations is shown in **Supplementary Figure S3**.

**Table 1:** Linear regression performance metrics.

Plant	Experimental type		Training		Testing		
		R²	Nitrate concentration (µg.g^-1^ DW)^*^	RMSE (µg.g^-1^ DW)	R²	Nitrate concentration (µg.g^-1^ DW)^*^	RMSE (µg.g^-1^ DW)
Amaranthus	Split Dataset	0.74	5.38 -> 28.16	3.8	0.61	8.35 -> 23.07	5.3
	LODO (Day 1 out)	0.60	7.96 -> 23.26	5.4	0.85	14.55 -> 31.83	2.9
	LODO (Day 2 out)	0.97	7.62 -> 26.70	1.3	0.46	7.77 -> 15.55	8.0
	LODO (Day 3 out)	0.73	8.60 -> 31.11	5.0	0.38	8.42 -> 22.96	3.7
Pak Choi	Split Dataset	0.93	97.53 -> 173.48	7.5	0.84	85.42 -> 173.99	11.5
	LODO (Day 1 out)	0.94	92.45 -> 168.04	8.2	0.91	128.12 -> 150.81	4.3
	LODO (Day 2 out)	0.99	98.04 -> 172.97	3.2	0.77	123.19 -> 169.76	13.6
	LODO (Day 3 out)	0.94	110.95 -> 167.51	5.4	0.85	80.41 -> 171.39	14.6

* Predicted mean nitrate concentration range.

For Pak Choi, the model was:


$$C_{i}=\;-708.90+846.17\;I_{i}$$

For Amaranthus, regression yielded:


$$C_{i}=\;-710.56+487.42\;I_{i}$$

where $C_{i}$is the predicted nitrate concentration (µg g^-^¹ FW) and $I_{i}$is the Raman peak ratio. Both models showed statistically significant relationships between Raman features and biochemical nitrate measurements (p = 0.030 for Pak Choi; p = 0.049 for Amaranthus).

**Supplementary Figure S3** illustrates the regression models used to convert normalized Raman peak ratios into quantitative nitrate estimates for both species, supporting the suitability of the selected spectral feature for nitrate quantification.

Cross-validation demonstrated model robustness. LODO validation yielded an RMSE of 38.2 µg g^-^¹ FW, while split-dataset validation produced an RMSE of 101 µg g^-^¹ FW with R² = 0.93 (training) and 0.84 (testing) for Pak Choi. For Amaranthus, LODO validation reflected lower absolute nitrate levels (+N ≈ 171 µg g^-^¹ FW), while split-dataset validation yielded R² = 0.74 (training) and 0.61 (testing) with RMSE = 32 µg g^-^¹ FW.

**Figure 3:**
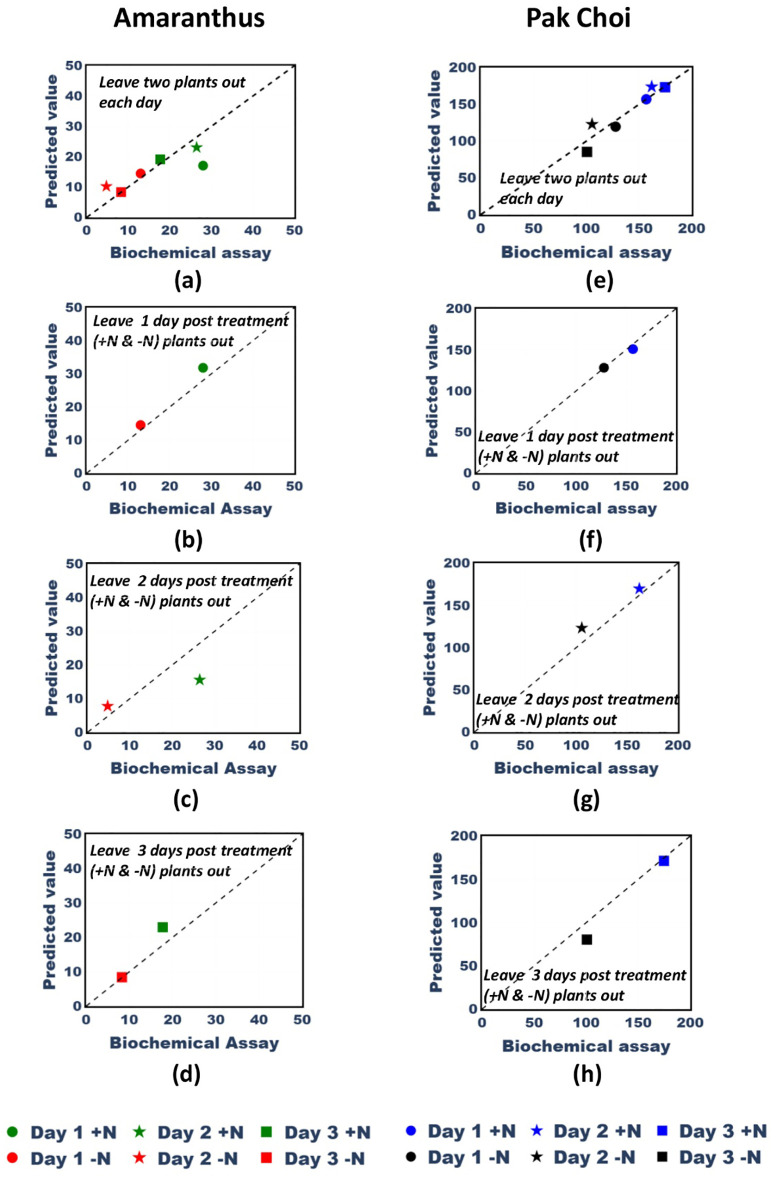
Prediction plots of median nitrate content in Amaranthus **(a–d)** and Pak Choi **(e–h)** based on a linear regression model. Cross-validation results: **(a, e)** - Model performance of 2 plants out under 1, 2, and 3 days of stress (Split Dataset). **(b–d, f–h)** Leave-One-Day-Out (LODO) - **(b, f)** Day 1 excluded; **(c, g)** Day 2 excluded; and **(d, h)** Day 3 excluded for Amaranthus and Pak Choi, respectively.

Differences in regression slopes (Amaranthus: 487.42; Pak Choi: 846.17) suggest species-dependent matrix effects, likely related to biomass composition and fresh-weight normalization. After dry-weight correction using literature ratios (Choi et al., 2023; Hwang et al., 2018), slopes converged (Amaranthus: 80.52; Pak Choi: 96.29), indicating improved comparability. The corresponding prediction plots are shown in **Figure 3**.

Comparative analysis with Partial Least Squares Regression (PLSR) and Support Vector Regression (SVR) confirmed that linear regression provides robust performance under current conditions (**Supplementary Tables 3**, **4**).

### 3.3 Integrating Raman spectroscopy with biochemical validation

The strong agreement between Raman-derived predictions and biochemical assays demonstrates that Raman spectroscopy provides a rapid, non-destructive approach for nitrate quantification in intact plant tissue. While biochemical assays were used for validation in this study, integration with complementary analytical techniques such as ion chromatography for direct nitrate measurement, HPLC-based metabolite profiling, and optical methods (chlorophyll fluorescence and UV–Vis spectroscopy) would further strengthen multi-modal validation across diverse stress conditions.

Collectively, these findings establish Raman spectroscopy as a reliable tool for early detection and quantitative monitoring of nitrogen status across C3 and C4 plant systems, with direct relevance to precision nutrient management in controlled-environment agriculture.

Conclusion

This study demonstrates that Raman spectroscopy enables rapid, non-destructive detection of nitrogen deficiency and quantitative estimation of nitrate content in Pak Choi (C3) and Amaranthus (C4). Spectral analysis captured early biochemical responses within 24 h of nitrogen withdrawal, preceding visible phenotypic changes.

A linear regression model based on the 1046/1067 cm^-^¹ Raman peak ratio accurately predicted nitrate concentration validated by biochemical assays. The model remained robust across species and stress progression, although species-specific differences in scaling were observed.

These findings establish Raman spectroscopy as a viable platform for real-time, quantitative plant nutrient monitoring and provide a foundation for future development of portable precision agriculture sensing systems.

## Data Availability

The raw data supporting the conclusions of this article will be made available by the authors, without undue reservation.
