# Supplementary material for: Raman spectroscopy enables non-destructive quantification of nitrate in C3 and C4 plants
Source: Front Plant Sci. 2026 Jul 27;17:1858997. doi: 10.3389/fpls.2026.1858997 (PMC13454117; doi:10.3389/fpls.2026.1858997)
Supplement: Supplementary file 1 [file DataSheet1.pdf]

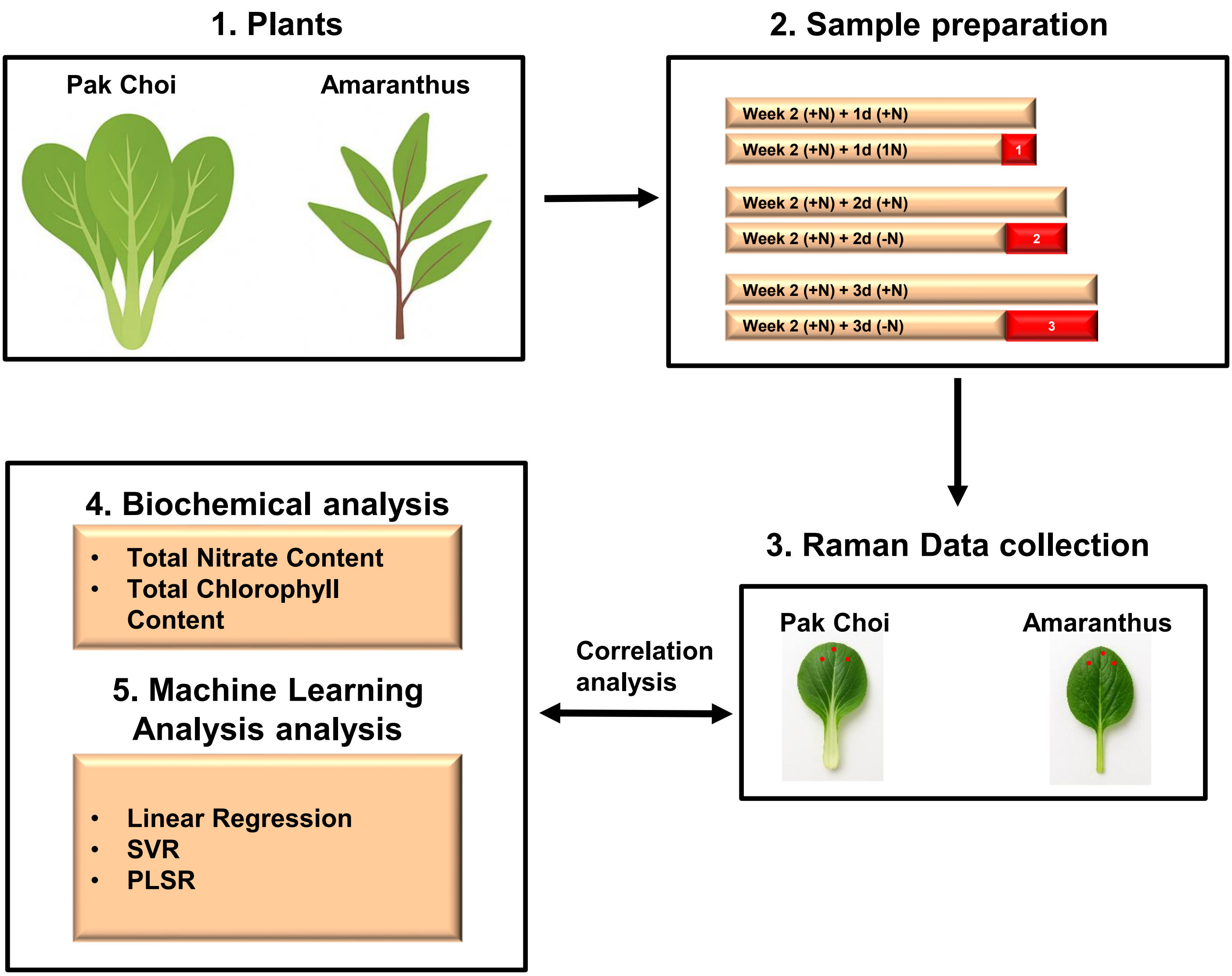

Supplementary Figure S1. Experimental workflow for nitrogen analysis in C3 (Pak Choi) and C4 (Amaranthus) plants.

Plants were grown under nitrogen-sufficient (+N) conditions for two weeks and subsequently transferred to nitrogen-deficient (–N) conditions for 1, 2, or 3 days. Leaf samples were analyzed for nitrate and chlorophyll content, and Raman spectra were collected from the adaxial leaf surface. The three red circles indicate representative positions used for spectral acquisition. Raman spectral data were correlated with biochemical measurements for validation. Pre-processed spectral data were further analyzed using machine learning models (Linear regression, SVR, PLSR) to estimate nitrate concentration, integrating physiological, biochemical, and spectral information to compare nitrogen responses between C3 and C4 species.

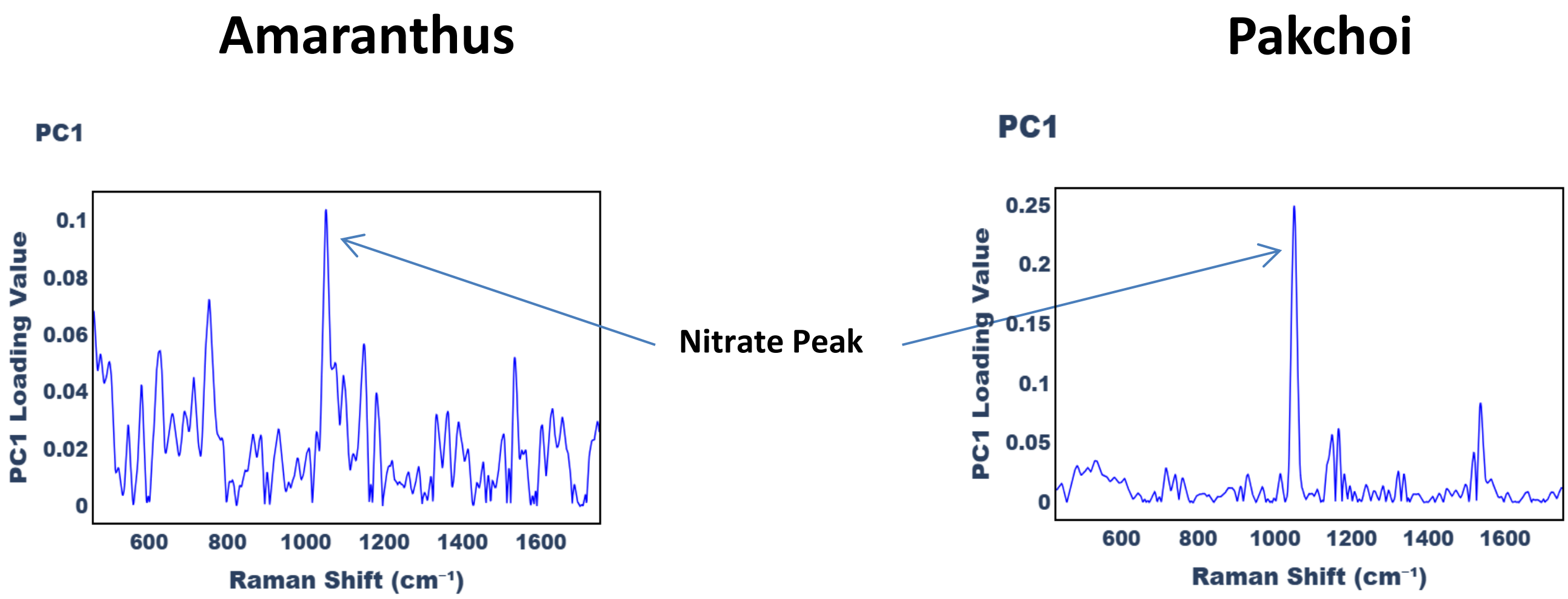

Supplementary Figure S2. Plots of the first Principal Component using all of the -N and +N data for Amaranthus and Pak Choi. The analysis clearly shows that the Raman peak at 1046 cm^{-1} is the primary change in the Raman spectra under nutrient deficient conditions.

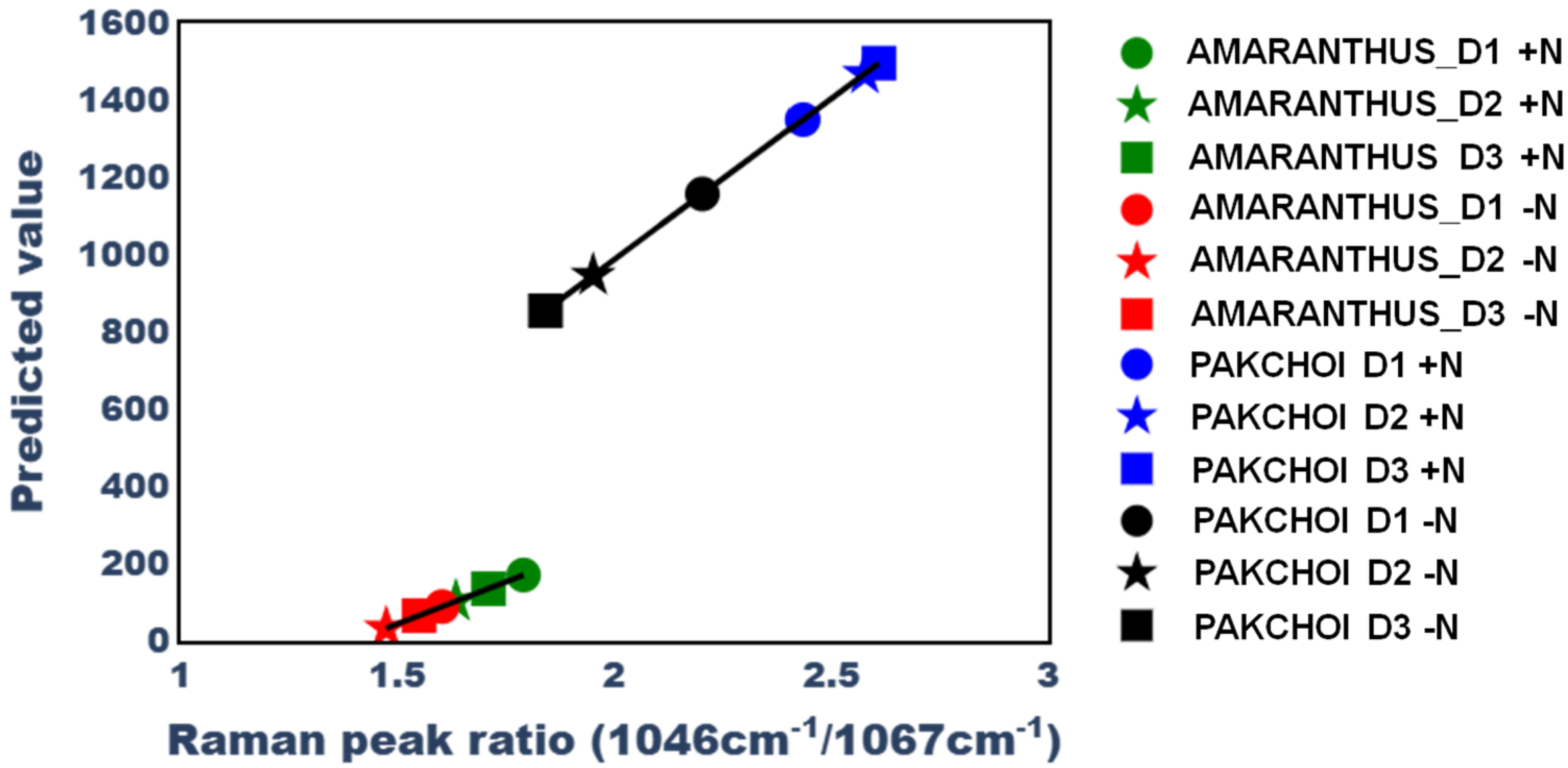

Supplementary Figure S3. Plots of the measured biochemical assay nitrate concentration (points) and the regression model ($C_i = \theta_o + \theta_1 I_i$) relating the nitrate concentration to the measured Raman peak ratio. The nitrate concentration has the units µg g^{-1} Fresh Weight (FW).

Supplementary Table 1: Assignment of Vibrational bands for the Raman spectra from plants

Raman peak (cm^{-1})	Vibrational mode	Assignment
480	C-C-O and C-C-C deformations. Related to glycosidic ring skeletal deformations.	Starch (Almeida et al., 2010)
520	ν(C-O-C) Glycosidic	Cellulose (Edwards et al., 1997; Agarwal, 2022)
747	γ(C–O–H) of COOH	Pectin (Sanchez et al., 2019; Farber et al., 2019)
917	ν(C-O-C) In plane, symmetric	Phenylpropanoids (Edwards et al., 1997)
1005	ν3 (C–CH3 stretching)	Carotenoids (Sanchez et al., 2019)
1046	NO_3 stretching	Nitrate (Gupta et al., 2020)
1155	C–C stretching; ν(C–O–C)	Carotenoids (Farber et al., 2019)
1320	δCH2 bending	Cellulose, Lignin (Sanchez et al., 2019; Farber et al., 2019)
1440–1446	δ(CH2) + δ(CH3)	Aliphatics (Yu et al., 2007)
1515–1535	–C=C– (in plane)	Carotenoids (Adar, 2017; Payne and Kurouski, 2021)
1606–1632	ν(C–C) Aromatic ring + σ(CH)	Phenylpropanoids (Payne and Kurouski, 2021)

Supplementary Table 2. *p*-values for Figure 1

**Plant**	**Model**	**Day**	***p*- value**
Amaranthus	Leaf area	Day 1	0.643
		Day 2	0.754
		Day 3	0.148
	Total Chlorophyll	Day 1	0.733
		Day 2	0.986
		Day 3	0.282
	Nitrate content	Day 1	6.348E-08
		Day 2	3.078E-10
		Day 3	8.492E-09
Pak Choi	Leaf area	Day 1	0.569
		Day 2	0.625
		Day 3	0.385
	Total Chlorophyll	Day 1	0.868
		Day 2	0.219
		Day 3	0.895
	Nitrate content	Day 1	4.620E-05
		Day 2	2.455E-10
		Day 3	9.021E-08

Supplementary Table 2. Model performance comparison for Split dataset

**Plant**	**Model**	**Training**		**Testing**	
		**R^2**	**RMSE ($\mu g.g^{-1}$ DW)**	**R^2**	**RMSE ($\mu g.g^{-1}$ DW)**
Amaranthus	Linear Regression	0.74	3.8	**0.61**	**5.3**
	PLSR	**0.93**	**1.1**	0.49	5.8
	SVR	0.67	4.1	0.32	6.7
Pak Choi	Linear Reg	0.93	**7.5**	**0.84**	**11.5**
	PLSR	**0.96**	**4.0**	0.60	**17.9**
	SVR	0.57	19.3	0.54	19.3

Supplementary Table 3. Model performance comparison for LODO

Plant	Test Days Out	Model	Training		Testing	
			R^2	RMSE ($\mu g.g^{-1}$ DW)	R^2	RMSE ($\mu g.g^{-1}$ DW)
Amaranthus	1	Linear Regression	**0.60**	5.4	**0.85**	**2.9**
	1	PLSR	**0.60**	**5.2**	0.18	6.8
	1	SVR	0.40	6.5	0.75	8.4
	2	Linear Regression	**0.97**	**1.3**	**0.46**	**8.0**
	2	PLSR	0.93	2.0	0.07	10.5
	2	SVR	0.51	4.4	0.24	11.0
	3	Linear Regression	**0.73**	**5.0**	0.38	**3.7**
	3	PLSR	0.65	5.6	0.03	3.7
	3	SVR	0.33	7.6	**0.42**	4.8
Pak choi	1	Linear Regression	**0.94**	**8.2**	**0.91**	**4.3**
	1	PLSR	0.76	16.3	0.10	14.5
	1	SVR	0.92	9.5	0.88	5.0
	2	Linear Regression	**0.99**	**3.2**	**0.77**	**13.6**
	2	PLSR	0.80	13.1	0.19	30.9
	2	SVR	0.98	3.67	0.71	15.2
	3	Linear Regression	**0.94**	**5.4**	0.85	14.6
	3	PLSR	0.70	12.5	0.25	32.0
	3	SVR	0.90	7.1	**0.95**	**8.4**
